# High-Pressure Microfluidic Homogenization Improves the Stability and Antioxidant Properties of Coenzyme Q10 Nanoliposomes

**DOI:** 10.3390/biology14050568

**Published:** 2025-05-19

**Authors:** Xinyu Li, Xingyu Zhao, Jing Wang, Baoshun Xu, Jin Feng, Wuyang Huang

**Affiliations:** 1 College of Food Science and Technology, Nanjing Agricultural University, Nanjing 210095, China; lixinyu6369@163.com; 2 Institute of Agro-Product Processing, Jiangsu Academy of Agricultural Sciences, Nanjing 210014, China; xzhwd0675@163.com; 3 College of Chemical Engineering, Nanjing Forestry University, Nanjing 210037, China; wjcrystal_gl@163.com; 4 Kangcare Bioindustry Co., Ltd., Nanjing 210006, China; xubaoshun@kangcare.com; 5 School of Food and Biological Engineering, Jiangsu University, Zhenjiang 212013, China

**Keywords:** coenzyme Q10, nanoliposome, high-pressure microfluidic homogenization, stability, antioxidant properties

## Abstract

Coenzyme Q10 is a natural antioxidant with anti-tumor and mitochondrial protective effects. However, its unstable physicochemical properties and large molecular weight result in low bioavailability. This study aimed to develop an effective technique for constructing nanoliposomes to improve the physicochemical properties of CoQ10 by using high-pressure microfluidic homogenization. Liposomes were prepared using the ethanol injection method and homogenized by high-pressure microfluidics to optimize their physicochemical properties. Liposome morphology and microstructure were observed via transmission electron microscopy (TEM). The particle size distribution, polydispersity index (PDI), and encapsulation efficiency were assessed, while effects on cell viability and antioxidant properties were investigated in HepG2 cells. The results indicate that the prepared liposomes exhibit favorable characteristics, including high encapsulation efficiency (>96%) and low PDI (<0.3), indicating uniform particle size distribution and good stability. The storage stability of liposomes at room temperature was significantly enhanced compared to liposomes not subjected to high pressure homogenization. In vitro cell experiments confirmed the liposomes’ non-cytotoxicity and substantial antioxidant activity, ensuring their safety for biomedical applications. This study introduced a liposome preparation method combining ethanol injection and high-pressure microfluidic homogenization, offering a novel approach for liposome modification with potential for development and application in innovative drug delivery systems and antioxidant therapy.

## Simple Summary

Coenzyme Q10 (CoQ10), an endogenous lipid-soluble antioxidant, is essential for cellular energy metabolism and oxidative defense. Although it shows great potential in treating cardiovascular and neurodegenerative diseases, its extremely low water solubility and bioavailability severely limit its clinical application. In this study, CoQ10 nanoliposomes prepared using the traditional ethanol injection method were optimized by high-pressure microfluidic homogenization technology. The optimized nanoliposomes exhibited uniform particle size distribution and excellent stability. The delivery system not only has no cytotoxicity but also can effectively protect HepG2 cells from oxidative stress damage, demonstrating significant antioxidant activity. High-pressure microfluidic homogenization successfully addressed the low encapsulation efficiency and stability issues of traditional liposome preparation. This advancement paves the way for the application of CoQ10 in precision medicine, particularly in targeted antioxidant therapy and mitochondrial protection. Furthermore, this platform can be expanded to develop delivery systems for other insoluble drugs.

## 1. Introduction

Coenzyme Q10, also known as ubiquinone, is a lipid-soluble antioxidant endogenously synthesized in humans and naturally present in foods such as fatty fish, organ meats, and whole grains [1]. As a critical component of the mitochondrial electron transport chain, CoQ10 facilitates ATP production during oxidative phosphorylation [2]. Beyond its role in energy metabolism, CoQ10 acts as a potent free radical scavenger, protecting cellular membranes, proteins, and DNA from oxidative damage while promoting cellular repair mechanisms [3]. Clinically, CoQ10 supplementation demonstrates therapeutic potential in cardiovascular diseases. It enhances myocardial bioenergetics, improves endothelial function, and alleviates symptoms of heart failure and hypertension [4,5]. Notably, cardiac patients exhibit significantly lower serum CoQ10 levels compared to healthy individuals. Statins, the first-line therapeutics for dyslipidemia, inhibit the mevalonate pathway [6], reducing endogenous CoQ10 synthesis, which may exacerbate muscle-related adverse effects [7]. However, CoQ10 supplementation mitigates statin-associated myopathy, reduces systolic blood pressure, and improves glycemic control in type 2 diabetes [8,9]. Despite dietary sources, endogenous production and food-derived CoQ10 often prove insufficient due to the molecule’s poor bioavailability [10]. While athletes utilize CoQ10 to enhance exercise tolerance and accelerate recovery through improved mitochondrial efficiency, its therapeutic application remains limited by physicochemical constraints [11]. As a high-molecular-weight, highly lipophilic compound, CoQ10 exhibits low aqueous solubility, instability under light/heat, and erratic gastrointestinal absorption. Emerging delivery systems, including liposomes and nanoemulsions, aim to enhance bioavailability, addressing current formulation challenges.

Liposomes are double-layered vesicles formed by the self-assembly of phospholipid molecules that possess unique characteristics of a hydrophilic core and a hydrophobic interlayer [12]. They can simultaneously encapsulate water-soluble (e.g., vitamin C) and lipophilic (e.g., coenzyme Q10) active ingredients, significantly improving their stability, bioavailability, and targeted delivery efficiency. As a representative of nanocarrier systems, liposomes protect encapsulated substances from enzymatic hydrolysis, oxidation, and other degradation during in vivo circulation [13]. Therefore, liposomes demonstrate significant potential in enhancing the stability and bioavailability of CoQ10 [14]. Liposome preparation technologies, including thin-film dispersion, ultrasonic processing, and ethanol injection, have been extensively studied to tailor liposome characteristics such as size, polydispersity index (PDI), and encapsulation efficiency, which affect their delivery [15,16]. Currently, the most widely studied liposome preparation methods include thin-film hydration and ethanol injection. Thin-film hydration is low-cost and simple to operate but time-consuming and primarily suited for hydrophilic drugs [16]. The ethanol injection method is a relatively simple and rapid method for preparing uniform liposomes that is suitable for large-scale production. However, liposomes prepared by this method may exhibit drug leakage, aggregation or fusion, and non-uniform size distribution, potentially compromising the encapsulated substance’s efficacy or pharmacokinetics. Therefore, it is necessary to develop a more efficient and economical preparation process for liposomes.

High-pressure microfluidic homogenization technology is a new dynamic homogeneous emulsification technology that enables the uniform mixing of oil and water phases for the preparation of liposomes and nanoemulsions [17]. It uses high-speed impact, high-frequency vibration, resultant tension shear, and ultra-high pressure to strongly shear the material; using this method, the droplets are extensively broken up and have an improved particle size distribution [18,19]. Ultra-high pressure microfluidic treatment causes structural changes in casein micelles and new casein cocoa interactions, thereby increasing the dispersion stability of cocoa in chocolate milk [20]. In addition, high-pressure microfluidic homogenization treatment can enhance nutritional quality by optimizing pressure parameters, such as increasing the β-carotene content in carrot juice by 530% when the juice is circulated three times at 68.95 MPa [21]. This treatment also helps maintain the antioxidant activity of sugarcane juice when treated at 150 MPa [22]. Romano et al. used high-pressure microfluidic homogenization to improve the structure of liposomes, which could enhance the storage stability and bioavailability of encapsulated vitamin C; this method is expected to achieve industrial expansion [23]. The low energy consumption characteristics of this method meet the requirements of modern green manufacturing and provide a new solution for the sustainable development of products.

In this study, CoQ10 liposomes were prepared using high-pressure microfluidic homogenization. The morphological characteristics, particle size distribution, PDI, zeta potential, encapsulation efficiency, and storage stability were analyzed. Additionally, HepG2 cell viability and the inhibitory effects of CoQ10 liposomes on H_2_O_2_-induced oxidative stress were evaluated. This study demonstrates the potential of high-pressure microfluidic homogenization in liposome preparation and provides foundational data for CoQ10 liposome production.

## 2. Materials and Methods

### 2.1. Chemical and Reagents

Coenzyme Q10 and phosphatidylcholine (egg yolk lecithin) were obtained from Xi’an Tiangyuan Biotechnology (Xi’an, China). Cholesterol was purchased from Ye Sen Biotechnology (Shanghai, China). Anhydrous ethanol, isopropanol, n-pentane, hydrogen peroxide, and phosphotungstic acid were bought from Sinopharm (Shanghai, China). HepG2 cells were purchased from CW Biotechnology (Beijing, China). Dulbecco’s modified Eagle medium (DMEM) was obtained from Gibco Biotechnology (Waltham, MA, USA). A dichloro-dihydrofluorescein diacetate (DCFH-DA) detection kit was obtained from Biyotime Institute of Biotechnology (Shanghai, China), and a cell counting kit-8 (CCK-8) kit was obtained from Vazyme Biotech (Nanjing, China). All reagents were of analytical grade.

### 2.2. Preparation of Liposomes

Liposomes were prepared via the ethanol injection method [24]. Phosphatidylcholine, cholesterol, and glycerol were combined in an optimized mass ratio (25:40:18, total 4.15 g) to form a lipid mixture, which was co-dissolved with CoQ10 (0.6 g) in 50 mL of ethanol preheated to 50 °C. This ethanolic solution was rapidly injected into 1 L of preheated aqueous phase (50 °C) under continuous magnetic stirring for 30 min. Ethanol was subsequently removed by rotary evaporation at 55 °C, yielding a crude liposomal aqueous dispersion. The dispersion was emulsified using an overhead stirrer (EUROSTAR 20 high speed digital, IKA, Bitterfeld-Wolfen, Sachsen-Anhalt, Germany) at 15,000 rpm for 5 min and ultrasonic instrument (KQ-100VDV, Kunshan Ultrasonic Instrument Co., Ltd., Kunshan, Jiangsu, China) at 360 W for 5 min, followed by maturation at 4 °C for 2 h to stabilize the bilayer structure. The pre-treated dispersion was subjected to a high-pressure microfluidic homogenizer (Microfluidics International Corporation, Westwood, MA, USA) under varying conditions (100 MPa × 1 cycle, 100 MPa × 3 cycles, 150 MPa × 1 cycle, and 150 MPa × 3 cycles) to refine particle size and obtain the final coenzyme Q10 nanoliposomes.

### 2.3. Characterization of Liposomes

#### 2.3.1. Transmission Electron Microscope (TEM) Observation

The coenzyme Q10 liposome sample was appropriately diluted and dropped onto the surface of a 300-mesh copper grid. After drying at room temperature, samples were negatively stained with 2% (*w*/*v*) aqueous phosphotungstic acid for 30 s. The microstructure was observed using an HT7800 transmission electron microscope (Hitachi Hi-Tech, Tokyo, Japan), with images captured at 80,000 × magnification.

#### 2.3.2. Dynamic Light Scattering (DLS) Measurement

A 10 mg amount of the CoQ10 liposome sample was diluted with 4 mL deionized water and equilibrated at room temperature for 30 min to avoid multiple scattering phenomena. The particle size, polydispersity index (PDI), and zeta potential were measured by a Nano particle potential analyzer (NICOMP Z3000, Malvern Panalytical, Malvern, UK) at room temperature.

#### 2.3.3. Entrapment Efficiency (EE)

For detecting total CoQ10, the nanoliposomes were combined with isopropanol in a ratio of 1:10 (*v*/*v*), vortexed vigorously for 3 min to disrupt liposomal structure, diluted 10-fold with deionized water, and adjusted to a final volume of 10 mL with isopropanol. The absorbance of the sample at 278 nm was measured and the total coenzyme Q1 content was calculated according to the standard curve.

For detecting free CoQ10, the liposomes were mixed with n-pentane in a ratio of 1:1 (*v*/*v*), vortexed for 3 min, and centrifuged at 2000 rpm for 20 min to separate the aqueous phase from the n-pentane phase. After removing the aqueous phase, the n-pentane was evaporated with nitrogen, and the residue was adjusted to a final volume of 10 mL with isopropanol. The absorbance of the sample at 278 nm was measured and the free coenzyme Q10 content was calculated according to the standard curve.

The EE of coenzyme Q10 in nanoliposomes were determined using the following equation:

$$Entrapment\;efficiency\;(\%)=\frac{\left(C_{1}-C_{2}\right)}{C_{2}}\times 100\%$$

where C_1_ is the total coenzyme Q10 content and C_2_ is the free coenzyme Q10 content.

### 2.4. Storage Stability

The nanoliposome samples prepared under different conditions were stored at 4 °C, room temperature (25 °C), and 37 °C. The encapsulation efficiency of CoQ10 nanoliposomes was detected after 20 and 40 days of storage, and the retention rate of CoQ10 was calculated using the following equation:

$$Retentiuon\;rate\;(\%)=\frac{{EE}_{n}}{{EE}_{1}}\times 100\%$$

where EE_1_ is the encapsulation rate on the first day and EE_n_ is the encapsulation rate on the nth day, *n* = 20 or 40.

### 2.5. Cell Culture and Treatment

HepG2 cells were cultured in DMEM supplemented with 10% fetal bovine serum and 1% penicillin–streptomycin. The cells were kept at 37 °C in a 5% CO_2_ atmosphere (MCO-20AIC incubator, Panasonic Healthcare, Osaka, Japan), and their morphology and growth were monitored daily. Upon reaching 80–90% confluency, the cells were subcultured to maintain them in the logarithmic growth phase. Four hours prior to the experiment, the medium was changed to a reduced-serum medium to induce a quiescent state in the cells.

#### 2.5.1. Cell Viability Assay

The adjusted value of the HepG2 cell suspension in logarithmic growth phase was 3 × 10^5^ cells/mL, which was transferred to a 96-well plate and incubated for 24 h (100 µL/well). Then, 100 µL of 150 MPa × 3-CoQ10 nanoliposome solution (0, 1, 3.125, 6.25, 12.5, 25, and 50 μg/mL) was added. After incubating the cells for 24 h, 10 µL of CCK-8 solution was added 1 h before the end of the incubation period, and the absorbance was measured at 450 nm. The control was cells without CoQ10 nanoliposome, whereas the blank was medium without cells. Cell viability was calculated using the following equation:

$$Cell\;viability\;(\%)=\frac{\left(A_{1}-A_{0}\right)}{{(A}_{2}-A_{0})}\times 100\%$$

where A_1_ is the absorbance of the CoQ10 nanoliposome sample, A_2_ is the absorbance of control group, and A_0_ is the absorbance of blank group.

#### 2.5.2. Reactive Oxygen Species (ROS) Assay

The ROS in HepG2 cells was assessed using the DCFH-DA detection kit. Logarithmically growing HepG2 cell suspensions (3 × 10^5^ cells/mL) were seeded in 12-well plates. After incubation for 24 h, 150 MPa×3-CoQ10 nanoliposome was added to achieve different concentrations (0, 1, 3.125, 6.25, 12.5, 25, and 50 μg/mL) and pretreated for 24 h. Then, 500 μM DMEM medium containing H_2_O_2_ was added to induce oxidative stress and the cells cultured for a further 24 h. After washing cells with PBS, 10 μmol/L DCFH-DA was added and reacted for 20 min at 37 °C before the cells were washed thoroughly with PBS. A group of cells was immediately visualized under an IX53 Inverted Fluorescent Microscope (Olympus, Tokyo, Japan) using 485 nm excitation and 530 nm emission filters. The images are presented at 200× magnification.

### 2.6. Statistical Analysis

Each set of experiments was performed in triplicate. Data were expressed as mean ± standard deviation, and error bars were plotted. The experimental data were analyzed using GraphPad Prism Version 8 (GraphPad Software, Inc., San Diego, CA, USA). A one-way/two-way analysis of variance (ANOVA) was performed to determine statistical differences among different groups. Statistical significance was defined as *p* < 0.05.

## 3. Results and Discussion

### 3.1. Microstructure of Coenzyme Q10 Nanoliposomes

**Figure 1:**
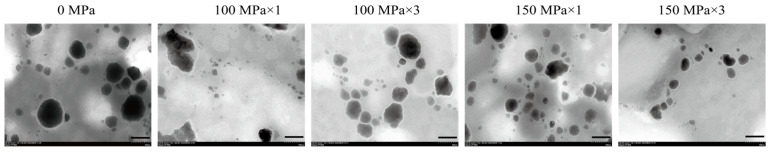
Transmission electron microscopy (TEM) image of coenzyme Q10 nanoliposomes (with high-pressure microfluidic homogenization treatment 0 MPa × 0 cycle, 100 MPa × 1 cycle, 100 MPa × 3 cycles, 150 MPa × 1 cycle, and 150 MPa × 3 cycles). Scale bar: 200 nm.

Under transmission electron microscopy (TEM), CoQ10 nanoliposomes predominantly exhibited an irregular spherical morphology (Figure 1). High-pressure microfluidic homogenization treatment significantly modified the particle characteristics. Increasing processing pressure and cycle number progressively reduced particle size while improving distribution homogeneity. This mechanical shear force induced lipid bilayer reorganization, resulting in smaller vesicles with enhanced dispersity [25]. The processed nanoliposomes maintained structural integrity the despite size reduction, showing intact and closed bilayer structures with surface irregularities attributable to CoQ10’s hydrophobicity influencing membrane packing [2,26].

### 3.2. Particle Size Distribution

Particle size serves as a critical determinant of liposomal performance, profoundly influencing both physicochemical properties and biological behavior [27]. Liposomes are strategically designed to have a size of less than 400 nm in order to passively accumulate in the tumor microenvironment through the enhanced permeation and retention effect [28]. Nanosized liposomes, typically with a particle size of less than 200 nm, are less likely to be recognized by macrophages, which can reduce immune rejection and prolong systemic circulation and the effective concentration period [29]. Smaller liposomes exhibit superior performance compared to their larger counterparts, demonstrating enhanced physical stability with reduced sedimentation and aggregation. Their increased surface area enables faster diffusion rates and an improved ability to traverse biological membranes, making them particularly suitable for intracellular drug delivery [11].

**Figure 2:**
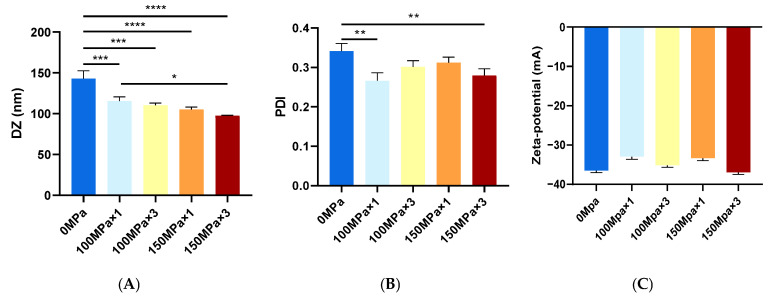
Particle size (**A**), polydispersity index (PDI, (**B**)), and zeta potential (**C**) of coenzyme Q10 nanoliposomes (with high-pressure microfluidic homogenization treatment at 0 MPa × 0 cycle, 100 MPa × 1 cycle, 100 MPa × 3 cycles, 150 MPa × 1 cycle, and 150 MPa × 3 cycles). Data are represented as mean ± standard deviation (SD, *n* = 3). Differences were considered significant at * *p* < 0.05, ** *p* < 0.01, *** *p* < 0.001, and **** *p* < 0.0001.

The Z-average size of all liposome samples prepared in this study was less than 150 nm (Figure 2A). Consistent with the TEM findings, high-pressure microfluidic homogenization treatment significantly reduced the liposome particle size. With increasing processing pressures and cycle numbers, the Z-average size of liposomes decreased progressively from 115.54 ± 5.17 nm (100 MPa × 1) to 110.47 ± 2.64 nm (100 MPa × 3), 105.26 ± 2.86 nm (150 MPa × 1), and 97.40 ± 0.62 nm (150 MPa × 3). All samples treated with high-pressure microfluidic homogenization exhibited a statistically significant size reduction compared to the untreated control (142.87 ± 9.72 nm at 0 MPa; *p* < 0.001). When liposomes were cycled three times at 150 MPa, the average particle size decreased by 31.83%, indicating the substantial influence of high-pressure microfluidic homogenization on liposome nanostructure dimensions.

The polydispersity index (PDI) serves as a critical metric for evaluating colloidal system uniformity, where values approaching 0.1 indicate near-monodispersed populations (<0.3 is generally acceptable) [30]. High-pressure homogenization technology demonstrated size-distribution refinement, as the PDI values reduced from 0.341 ± 0.019 in the control group to 0.266 ± 0.020 (100 MPa × 1, *p* < 0.01), 0.299 ± 0.015 (100 MPa × 3), 0.292 ± 0.014 (150 MPa × 1), and 0.279 ± 0.017 (150 MPa × 3, *p* < 0.01, Figure 2B). After high-pressure microfluidic homogenization treatment, all the PDI values of the CoQ10 nanoliposomes were less than 0.3, which represents an acceptable stable dispersion. The high-pressure microfluidic homogenization process enhances the homogeneity of nanoliposomes through turbulent shear forces disrupting vesicle aggregation, cavitation-induced lipid bilayer reorganization, and energy-dependent redistribution of particle size [31]. The limited PDI reduction may reflect the intrinsic mechanical stability of cholesterol-rich bilayers, which resist further homogenization-driven size refinement. Future studies could systematically vary lipid composition to decouple these effects. After high-pressure homogenization optimization, the particle size of nanoliposomes decreases, the specific surface area increases, and the drug loading can be increased. The synergistic application of ethanol injection and high-pressure microfluidic homogenization techniques enables a precisely controlled, nanoscale fabrication process for CoQ10-loaded nanoliposomes. This approach enhances the stability and uniformity of the processed samples, thereby improving the storage stability of the CoQ10 nanoliposomes.

### 3.3. Zeta Potential

Figure 2C demonstrates that CoQ10-loaded nanoliposomes exhibited a zeta potential of less than −30 mV, confirming their moderate-to-strong negative surface charge. The negative zeta potential provided electrostatic repulsion between coenzyme Q10 nanoliposomes, which could help to prevent aggregation and improve colloidal stability, prolong systemic circulation time, and optimize transdermal drug delivery efficiency through charge-mediated skin interactions [32,33,34,35]. Notably, high-pressure microfluidic homogenization treatment showed no statistically significant alteration in surface charge (*p* > 0.05 vs. untreated liposomes), indicating preservation of electrostatic properties during the size reduction processes. Electrostatic repulsion between negatively charged liposomes effectively prevents aggregation and gravitational sedimentation, thereby enhancing their stability. The negative charge reduces nonspecific protein adsorption and subsequent macrophage clearance, prolonging systemic circulation [36]. Additionally, negatively charged liposomes penetrate the skin more easily [37]. Coenzyme Q10 can promote the renewal and repair of epithelial cells and granulation tissue to enhance the skin’s natural barrier and can also inhibit the activity of tyrosine phosphatase to achieve whitening and spot-removal effects [38]. Since negatively charged liposomes penetrate the skin barrier more easily, the liposomes developed in this study have potential applications in the cosmetics industry.

### 3.4. Liposome Encapsulation Efficiency

**Figure 3:**
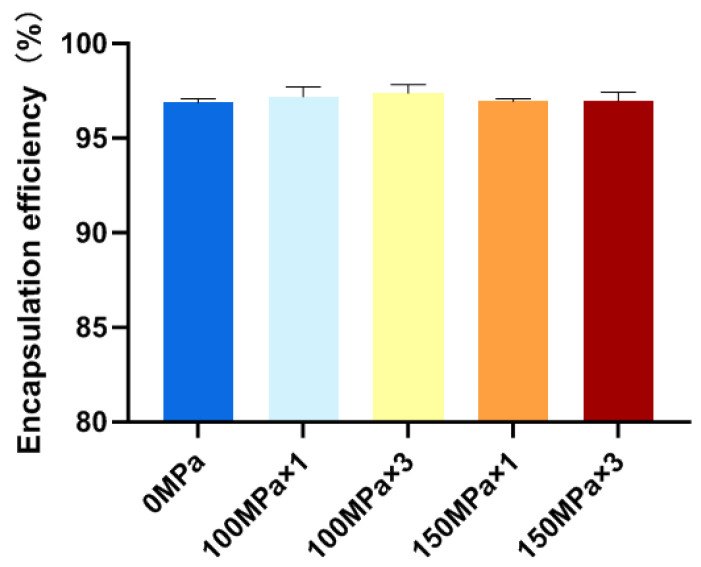
Comparison of the encapsulation efficiency of coenzyme Q10 nanoliposomes (with high-pressure microfluidic homogenization treatment at 0 MPa × 0 cycle, 100 MPa × 1 cycle, 100 MPa × 3 cycles, 150 MPa × 1 cycle, and 150 MPa × 3 cycles). Data are represented as mean ± standard deviation (SD, *n* = 3).

The liposome encapsulation efficiency is the ratio of active components encapsulated in liposomes during their preparation. It is one of the important parameters in evaluating the quality of liposome preparations, and the encapsulation efficiency measurement is an important means of liposome quality adjustment, which has great significance for controlling the amount of drug administered. As shown in Figure 3, high-pressure-microfluidic-homogenization-treated samples were not statistically significantly different to the untreated control samples (*p* > 0.05), and all CoQ10-loaded nanoliposomes exhibited a high encapsulation efficiency, with values above 96%, confirming their successful encapsulation. As the basic material for the construction of liposomes, phospholipids have amphiphilic molecular properties that make them an ideal carrier for drug encapsulation [39]. Lipophilic drugs can be trapped in the space between the lipid layers, and coenzyme Q10 shows a significant affinity with the phospholipid bilayer due to its strong lipophilicity, which explains the prepared liposome’s high encapsulation efficiency [40]. With or without high-pressure microfluidic homogenization, the liposome encapsulation efficiency can reach more than 96%. However, high-pressure microfluidic homogenization mainly affects the microstructure of the CoQ10 liposomes.

### 3.5. Liposome Storage Stability

**Figure 4:**
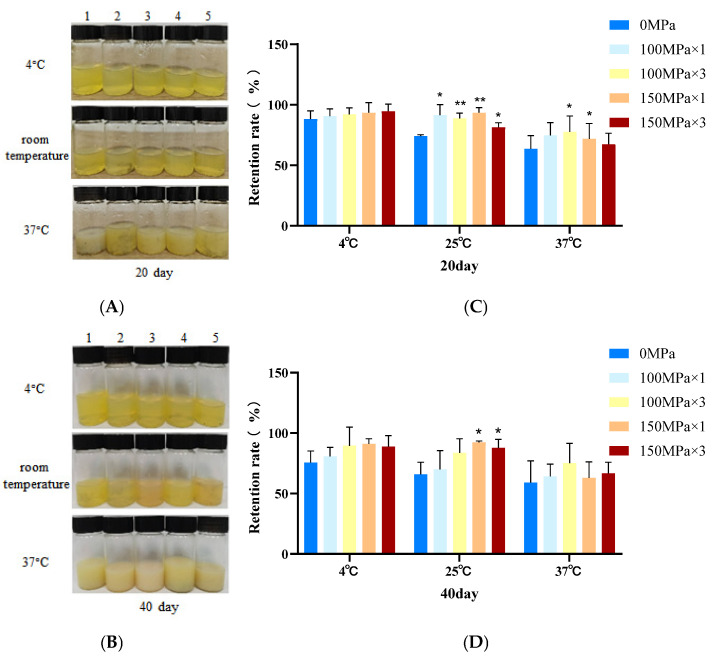
Visual appearance (**A**,**B**) and retention rate (**C**,**D**) of coenzyme Q10 nanoliposomes stored at different temperatures (4 °C, room temperature, and 37 °C) for 20 days and 40 days. From left to right (1, 2, 3, 4, 5), the photos are coenzyme Q10 nanoliposomes with high-pressure microfluidic homogenization treatment 0 MPa × 0 cycle, 100 MPa × 1 cycle, 100 MPa × 3 cycles, 150 MPa × 1 cycle, and 150 MPa × 3 cycles. Data are represented as mean ± standard deviation (SD, *n* = 3). Differences were considered significant at * *p* < 0.05 and ** *p* < 0.01 vs. the control (0 MPa).

Storage stability testing demonstrated temperature-dependent structural changes in CoQ10 nanoliposomes. Visual observation of the changes in appearance of CoQ10 nanoliposomes showed that optical clarity was maintained at 4 °C for 20 and 40 days, suggesting a preserved bilayer structural integrity without aggregation (Figure 4A,B). This stability is attributed to cholesterol’s ability to suppress phosphatidylcholine phase transition and maintain membrane rigidity [41]. In contrast, flocculation occurred at room temperature (25 °C), resulting from increased membrane fluidity due to the enhanced thermal movement of lipid molecules and weakened hydrophobic interactions [42]. At 37 °C, accelerated degradation manifested as emulsification, indicating bilayer disruption, CoQ10 leakage, and phospholipid reorganization into emulsion droplets during gel-to-liquid crystal transition [43].

The high-pressure microfluidic homogenization parameters significantly affected the stability results, with precipitate formation reduced at all temperatures. The retention of nanoliposomes further demonstrates a direct relationship between the storage stability, processing parameters (pressure magnitude and number of cycles), and colloidal stability enhancement [44]. Obviously, the effect of short-term storage at 4 °C for 20 days was the best, and the retention rate of nanoliposomes treated at 150 MPa three times reached 94.69 ± 5.85%. After 20 days at room temperature, the retention rate of CoQ10 nanoliposomes treated by high-pressure microfluidic homogenization was significantly better than that of the control group (*p* < 0.05, Figure 4C). Microfluidically processed formulations maintained structural integrity for 40 days at 4 °C and partial organization at room temperature but not at 37 °C. Notably, it can be found that the retention rate of CoQ10 nanoliposomes treated at 150 MPa was still significantly higher than the control’s retention rate, with an extension of storage time except at 25 °C (*p* < 0.05, Figure 4D). Consistent with the PDI results, it was found that the particle size was reduced by shear stress under high-pressure microfluidic homogenization and that the bilayer structure of nanoliposomes was stabilized during low temperature aging. Therefore, the CoQ10 nanoliposome samples were suitable for refrigerator storage.

Temperature is a more critical driver of degradation than the production process. In this study, physical optimization was used to improve the stability of CoQ10 nanoliposomes. Similarly, high-pressure microfluidic homogenization techniques are commonly used in milk processing to reduce fat separation and improve texture and taste. Complementary formulation strategies could further enhance stability. Chen et al. added Tween 80 to create steric hindrance by adsorbing onto the liposome surface, inhibit flocculation, prevent the thermal degradation of curcumin, and balance storage stability and membrane permeability [45]. Potato protein (PP) and soybean soluble polysaccharides (SSPSs) can also be used as carriers to encapsulate liposomes through electrostatic interaction in order to form multilayer emulsion structures and improve stability [46]. By providing a chemical protective barrier through formulation modification, synergistic treatment of the two may further promote the development of liposome technology and provide ideas for the design of other hydrophobic drug delivery systems. Physical experiments showed that nanoliposomes treated at 150 MPa for three cycles had good characteristics. In order to realize industrial production, further cell experiments were carried out to study their cytotoxicity and biological activity.

### 3.6. Antioxidant Properties in HepG2 Cells

Based on comprehensive stability assessments, CoQ10 nanoliposomes processed through three cycles of 150 MPa high-pressure microfluidic homogenization were selected for subsequent cell experiment investigations due to their exceptional physical stability and structural preservation capabilities.

**Figure 5:**
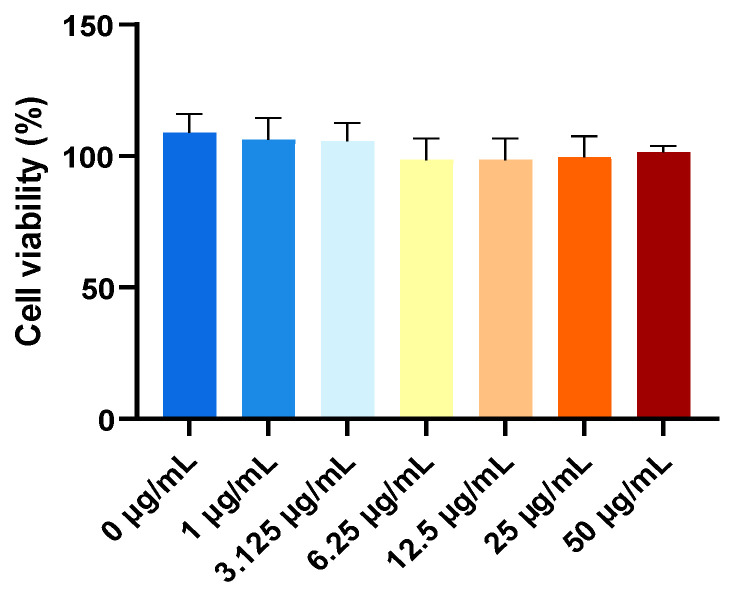
Cell viability of HepG2 cells treated with different concentrations of 150 MPa × 3-coenzyme Q10 nanoliposomes (0, 1, 3.125, 6.25, 12.5, 25, and 50 μg/mL). Data are represented as mean ± standard deviation (SD, *n* = 3).

**Figure 6:**
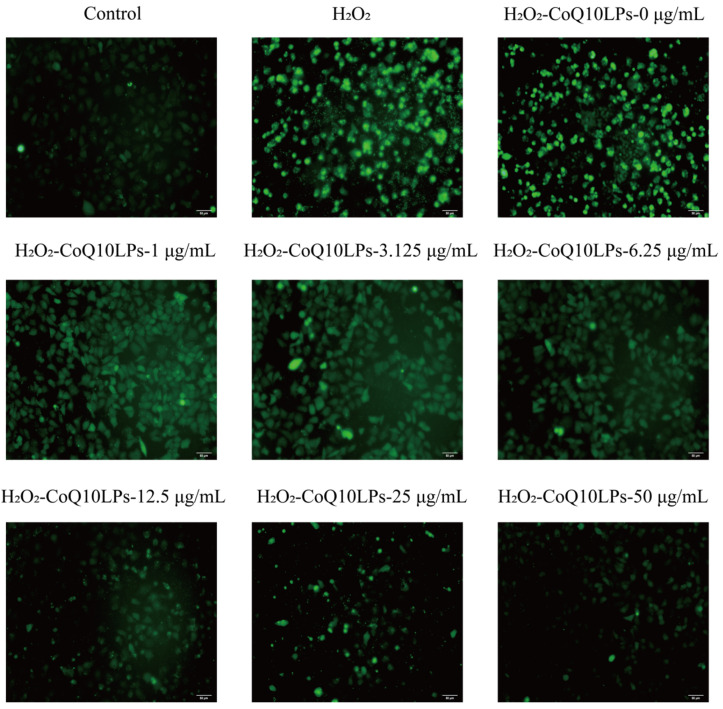
Effect of different concentrations of 150 MPa×3-coenzyme Q10 nanoliposomes (CoQ10LPs 0, 1, 3.125, 6.25, 12.5, 25, and 50 μg/mL) on H_2_O_2_-induced ROS levels in HepG2 cells. A representative set of fluorescence intensity images from three independent experiments is shown. All images presented are in 200× magnification.

Coenzyme Q10 nanoliposomes had no cytotoxicity, since there was no significant change between HepG2 cells treated with different concentrations of 150 MPa × 3-CoQ10 nanoliposomes and the control group (0 μg/mL) and all samples exhibited a high cell viability of approximately 100% (Figure 5). Figure 6 shows that H_2_O_2_ exposure induced a marked elevation in intracellular ROS levels, establishing an oxidative stress model in HepG2 cells. Notably, the 150 MPa × 3-CoQ10 nanoliposomes demonstrated concentration-dependent attenuation of fluorescence intensity, revealing their potent capacity to counteract H_2_O_2_-triggered oxidative damage. These findings confirm that the high-pressure-microfluidic-homogenization CoQ10 nanoliposomes developed in this study exhibit significant antioxidant properties through ROS scavenging mechanisms. After high-pressure treatment, the size of nanoliposomes decreases, making them more conducive to cell absorption through endocytosis and playing an antioxidant role. It is speculated that nanoliposomes can protect CoQ10 from oxidative degradation and preserve its biological activity in its reduced form, thereby sustaining its antioxidant capacity [47]. Oxidative stress and mitochondrial dysfunction exhibit a bidirectional pathological interplay. The free radicals generated by oxidative stress target mitochondrial complex I, impairing electron transport chain function. This damage subsequently exacerbates electron leakage, thereby further amplifying ROS production and establishing a self-perpetuating cycle of mitochondrial deterioration [48]. Mitochondrial dysfunction has been conclusively implicated in various neurodegenerative diseases, such as Parkinson’s disease and Alzheimer’s disease [49]. Therefore, subsequent studies should investigate the in vivo delivery and absorption kinetics of CoQ10 nanoliposomes using animal models to elucidate their bioavailability and underlying mechanisms of action. The blood–brain barrier (BBB) is a barrier composed of tightly connected endothelial cells that protects the central nervous system. However, due to its high selectivity, many drugs cannot penetrate it, posing a challenge for the treatment of central nervous system diseases. Significantly, engineered lipid-based nanoparticles (<200 nm) demonstrate enhanced BBB penetrability [50] and anti-inflammatory/antioxidant capacities [51,52,53]. To further validate the biological efficacy of CoQ10 nanoliposomes, in vitro BBB models should be established to investigate whether the nanoliposomes can break through the barrier or possibly restore mitochondrial redox homeostasis by delivering exogenous ubiquinone. This strategic supplementation may compensate for endogenous CoQ10 depletion, effectively disrupting the ROS mitochondrial damage cycle and attenuating neurodegenerative progression.

## 4. Conclusions

This study successfully prepared highly stable CoQ10 nanoliposomes using ethanol injection combined with high-pressure microfluidic homogenization technology. The experimental results demonstrated that the optimized nanodelivery system displayed exceptional physical and chemical characteristics, a highly uniform particle size distribution (PDI < 0.3), encapsulation efficiency exceeding 96%, and over 90% retention of the active ingredient after 40 days of storage at 4 °C. Transmission electron microscopy revealed that the nanoliposomes had a regular spherical structure with an average particle size below 150 nm. Additionally, the delivery system exhibited negligible toxicity towards HepG2 cells and effectively mitigated oxidative stress induced by H_2_O_2_. The stability and uniformity of coenzyme Q10 can be significantly improved through the high-pressure microfluidic homogenization process; however, the resulting product’s in vivo effect still needs to be studied, as does its applicability to industrial production.

## Data Availability

The data presented in this study are available upon request.
